# Plant-Derived Antimicrobial Peptides as Potential Antiviral Agents in Systemic Viral Infections

**DOI:** 10.3390/ph14080774

**Published:** 2021-08-06

**Authors:** Nour Mammari, Ysaline Krier, Quentin Albert, Marc Devocelle, Mihayl Varbanov, on behalf of the OEMONOM

**Affiliations:** 1 L2CM, Université de Lorraine, CNRS, F-54000 Nancy, France; nour.mammari@univ-lorraine.fr; 2 Faculté de Pharmacie, 7 Avenue de la Foret de Haye, 54505 Vandoeuvre-Les-Nancy, France; ysaline.krier9@etu.univ-lorraine.fr; 3 Fungal Biodiversity and Biotechnology, INRAE/Aix-Marseille University, UMR1163, 13009 Marseille, France; quentin.ALBERT@univ-amu.fr; 4 CIRM-CF, INRAE/Aix Marseille University, UMR1163, 13009 Marseille, France; 5 SSPC (SFI Research Centre for Pharmaceuticals), V94T9PX Limerick, Ireland; mdevocelle@rcsi.ie; 6 Department of Chemistry, Royal College of Surgeons in Ireland, RCSI University of Medicine and Health Sciences, 123, St. Stephen’s Green, D02 YN77 Dublin 2, Ireland

**Keywords:** antiviral peptides, systemic infection, virus, natural compounds, plants

## Abstract

Numerous studies have led to a better understanding of the mechanisms of action of viruses in systemic infections for the development of prevention strategies and very promising antiviral therapies. Viruses still remain one of the main causes of human diseases, mainly because the development of new vaccines is usually challenging and drug resistance has become an increasing concern in recent decades. Therefore, the development of potential antiviral agents remains crucial and is an unmet clinical need. One abundant source of potential therapeutic molecules are plants: they biosynthesize a myriad of compounds, including peptides which can have antimicrobial activity. Our objective is to summarize the literature on peptides with antiviral properties derived from plants and to identify key features of these peptides and their application in systemic viral infections. This literature review highlights studies including clinical trials which demonstrated that plant cyclotides have the ability to inhibit the growth of viruses causing human diseases, defensin-like peptides possess anti-HIV-1 activity, and lipid transfer proteins and some lectins exhibit a varied antimicrobial profile. To conclude, plant peptides remain interesting to explore in the context of emerging and re-emerging infectious diseases.

## 1. Introduction

First-generation antiviral molecules, such as amantadine, rimantadine, vidarabine, vidarabine phosphate, acyclovir and ribavirin, have serious side effects on humans due to their poor specificity [1]. Amantadine and rimantadine are useful for the treatment and prophylaxis of viral influenza A infections [1]. Vidarabine is the first drug to have become generally available in the USA for parenteral treatment of life-threatening or debilitating herpes simplex encephalitis, neonatal herpes simplex types 1 and 2, and varicella-zoster infections. It is an adenosine analog used as a replication inhibitor which can affect not only viral deoxyribonucleic acid (DNA) polymerase, but also the eukaryotic analog [2,3]. The advance of research in this area has led to the description of better molecules. Acyclovir, the first nucleoside analog and antiviral drug, is utilized for the treatment of herpes simplex virus (HSV) infections, particularly in genital herpes, and herpes zoster in varicella-zoster virus (VZV) infections [4]. This molecule causes lower toxicity to the host when compared with previously used treatments. Systemically administered ribavirin and aerosolized ribavirin are indicated for treating Lassa fever and respiratory syncytial virus (RSV) pneumonia in children and infants, and influenza A infections in adults, respectively [5,6,7]. Unfortunately, the low efficacy of antiviral treatments is still evidenced by the ever-increasing reports of viral resistance [6].

In addition, antiviral agents, including inhibitors of viral DNA polymerase, such as the nucleoside analog ganciclovir, the nucleotide analog cidofovir, and the pyrophosphate analog Foscarnet target mostly active CMV infections [7]. All these drugs are limited in their efficacy and have dose-related toxicities against CMV virus [8].

Other antiviral strategies are used to fight emerging viral infections. Vaccination is one of the most effective methods to protect against seasonal influenza virus-related epidemics. However, seasonal vaccines vary in efficacy; they can be ineffective in the elderly population and they do not provide protection against novel strains. These strains have developed resistance to neuraminidase inhibitors and nearly complete resistance to M2 ion channel inhibitors, the latter being part of the influenza envelope surface proteins [9]. There are several combination chemotherapy treatments for human immunodeficiency virus (HIV) infection. These anti-HIV therapies have led to the development of HIV resistance to drugs. Even with combination chemotherapy, resistance is still a major problem. Nevertheless, resistance patterns tend to change over time. Recent developments of resistance to several new anti-HIV drugs have been observed, including resistance to newer non-nucleoside reverse transcriptase inhibitors, integrase inhibitors, and C–C chemokine receptor type 5 (CCR5) antagonists (e.g., maraviroc), which block a host co-receptor. It should be noted that there are molecular differences in resistance mechanisms, even with drugs of the same class. Although advances in anti-HIV therapy continue, the virus keeps evolving and developing new mechanisms of resistance to anti-HIV drugs [10]. Indeed, the phenomenon of direct-acting antiviral drug resistance in the hepatitis C virus (HCV) has become increasingly alarming. This has led to considerable interest in identifying common resistance-associated mutations and in understanding the biochemical mechanisms underlying viral resistance [11,12].

In this alarming context, recent evidence reveals that some antimicrobial peptides may have activity against a broad range of viruses. Plant-derived defensive peptides have become the focus of numerous studies for their potential use as novel molecules in the treatment of human viral diseases [13]. For instance, cyclotides are a large family of plant-derived peptides that have a broad range of biological roles, including antimicrobial, anthelminthic, nematocidal, and insecticidal activities [14]. Cyclotides from different plant species have been significantly investigated for their ability to inhibit the growth of viruses that are involved in human diseases by disrupting the viral envelope [15], as in the case of HIV, influenza virus H1N1 and dengue virus (DENV). Other plant defense-related peptides may curtail human virus infections by interacting and interfering with proteins and enzymes that are fundamental for the viral replication cycle [16] Among these peptides, plant defensins have predominantly been investigated against fungal pathogens and some bacterial strains, but molecules which derive from defensin also possess the capacity to reduce the activity of HIV-1 reverse transcriptase [16,17]. Furthermore, a number of lectins present antiviral activity and their use has been mostly suggested as antiretroviral microbicides [18]. Lectins can bind to virions and prevent viral fusion and entry into target cells, thereby preventing infection [18]. Some of the most promising anti-HIV lectins include griffithsin (GRFT) [19], banana lectin (BanLec) [20], *Artocarpus heterophyllus* lectin (jacalin) [21], *Canavalia ensiformis* lectin [22], *Galanthus nivalis* (snowdrop) agglutinin-related lectins [23], *Myrianthus holstii* lectin [24], *Narcissus pseudonarcissus* lectin [25], *Polygonatum cyrtonema* lectin (PCL) [26], and *Boodlea coacta* lectin [27] among others. Based on published evidence, plant-derived peptides have antiviral activity against a wide range of human viral infections such as HIV, Middle East Respiratory Syndrome coronavirus (MERS-CoV), severe acute respiratory syndrome coronavirus (SARS-CoV), HCV, HSV-2, human papillomavirus (HPV), DENV, Ebola virus (EVD) and Alphaviruses (Table 1). The main objective of this literature review is to provide an overview of plant-derived peptides exhibiting antiviral activity on human systemic infections.

## 2. Plant-Derived Antiviral Peptides (AVP) against DNA Viruses

### 2.1. Herpes Simplex Virus 1 and 2 (HSV)

In 2016, more than 3.7 billion people under 50 years of age were infected by herpes simplex virus-1 (HSV-1) and almost 491 million people between the ages of 15 and 49 were infected by herpes simplex virus-2 (HSV-2) [37]. After a primary severe infection, these two viruses establish latency into sensory ganglia and cause latent infections and reactivations leading to cutaneous, oral or genital herpes, keratitis, conjunctivitis and encephalitis, thus affecting quality of life. No curative treatment is currently available [38]. The lipid bilayer envelope with glycoproteins of the viral particle protects the protein matrix called tegument, which protects, in turn, the 162 capsomeres of the icosahedral capsid [38]. Inside, the viral genome consists of a double-stranded DNA core encoding many enzymes, with more than 80 proteins and 90 transcriptional units for HSV-1 alone [38]. Replication and assembly of each new viral particle generation result in host cell death [38]. Prevention and prophylaxis are the main therapeutic strategies based on the use of microbicides and vaccines, along with symptomatic treatment [39].

Isolated from *Sorghum bicolor* seeds (Poaceae family—mostly found in Africa [40]), 2 kD peptide is a cationic, amphiphatic peptide that exerts a HSV-1 virucidal activity by targeting viral replication cycles [41]. Even if its mechanisms of action are not perfectly understood, this peptide seems able to interact with viral envelope proteins by binding or masking them [41]. One study explained that after inoculation of Vero cells with HSV-1, the peptide showed a dose-dependent action by reducing the virus replication by 40–90% [42]. The half maximal effective concentration of the peptide (EC_50_) is 6.25 μM, the 90% maximal effective concentration (EC_90_) value is 15.25 μM and the value of half maximal inhibitory concentration of the peptide required for inhibition of infection (IC_50_) is 250 μM [42]. This peptide also showed a dose-dependent action against HSV-1-induced cytopathic effects. Its inoculation in Vero cells before infection with HSV-1 resulted in an EC_50_ value of 12.25 μM, whereas values of EC_50_ are 6.25 μM when the peptide is inoculated on cells during and after infection with HSV-1. Other experiments regarding incubation of the peptide with other viruses led to the conclusion that this peptide may also have an in vitro prophylactic effect against HIV-1 [42].

Some defensins use the same supposed mechanism as the previous peptide: these small (around 50 amino-acids, at maximum) cationic and cysteine-rich peptides protect the host by binding viral glycoproteins before their interaction with host cells [42,43].

Meliacine is a glycopeptide isolated from leaves of *Melia azedarach L*., also called “Chinaberry tree”, from the Meliaceae family—native to Asia, but also found in America, Northern Australia, Africa and Southern Europe [43]. Meliacine targets the replication cycle of HSV-1 and, more specifically, polypeptides participating in viral DNA synthesis and in the assembly of viral nucleocapsids in the infected cell. The treatment of HSV-1-infected Vero cells with 50 μg/mL of meliacine leads to a reduction of 45% in the quantity of the viral DNA, disruption of the spread of viral particles and of the proportion of mature virus particles [44,45].

In an in vivo study, the peptide was administrated topically during 4 days to Balb/c mice infected by HSV-1 inducing a herpetic stromal keratitis. The results showed that meliacine significantly reduced histological damage to the cornea and reduced clinical signs. Compared to control mice, the quantity of isolated virus was 2-fold lower. No toxic effects to host cells due to this peptide have been identified [45]. Another study confirms the efficiency of meliacine in inhibition of the viral replication of a wild-type HSV and a thymidine kinase-deficient HSV-1 mutant (TK^−^) (EC_50_ of 0.82 and 0.41 μg/mL, respectively), without alterations to host cell viability, and suggests the use of this peptide, and the action of acyclovir, for a synergistic effect against HSV-1 replication [46].

The action of this peptide has been tested against HSV-2 with topical administration of 15.00 ± 1.93 mg/mL of meliacine to Balb/c mice infected by HSV-2 inducing a genital herpetic infection. Results showed that meliacine increased host survival, and reduced by 90% the severity of clinical symptoms and the presence of viral particles in vaginal fluids. It is noteworthy that the composition of this fluid was different from the one of untreated mice, as it presented increasing amounts of IFN-gamma (2550 ± 63 pg/mL) and TNF-alpha (from 7 ± 0.9 ng/mL to 12.15 ± 0.92 ng/mL), cytokines implicated in inflammation [47,48]. This ability to increase cytokine production has been confirmed by another study using 56 µg/mL of meliacine applied in lipopolysaccharide-producing *Escherichia coli* bacterial infection [47,48].

### 2.2. Human PapillomaVirus (HPV)

Human papilloma viruses (HPV) are a small group of non-enveloped viruses belonging to the Papillomaviridae family. The viral particles consist of a genome in the form of a circular double-stranded DNA. HPV infection remains the second most frequently occurring cause of cervical cancer in women worldwide, with an estimated 291 million HPV-positive women in 2007 [72]. The incidence and prevalence of this pathology vary mostly on factors including HPV genotype [73]. So far, there only preventative HPV vaccines that have been endorsed by the United States Food and Drug Administration (FDA), namely Cervarix^®^ and Gardasil^®^ These vaccines are accessible for early prophylaxis of infection with common cancer-causing HPV types, but local and systemic treatments are still under review [74].

The preclinical evaluation of griffithsin (GRFT), a lectin isolated from the red marine alga Griffithsia sp., has been identified to be an antiviral compound against human papillomavirus (HPV) in vitro with an EC_50_ 35.1 µg/mL (1.39 µM) [29]. Another study evaluated the anti-HPV activity of GRFT and found an EC_50_ of 10.8 µg/mL (0.428 µM) for HPV18, and an EC_50_ of 23.4 µg/mL (0.928 µM) for HPV45 [30].

## 3. Plant-Derived Antiviral Peptides against RNA Viruses

### 3.1. Human Immunodeficiency Virus (HIV)

HIV-1 and HIV-2 belong to the family of Retroviridae and genus. They are enveloped RNA viruses and rely on the enzyme reverse transcriptase to transcribe their genome from RNA into DNA, which can then be integrated into the host’s genome with an integrase enzyme, becoming part of the cellular DNA and replicating with it [75].

HIV was first isolated in 1983. Transmission can be horizontal (blood, sexual transmission) or vertical (mother to child through pregnancy). The virus targets mainly T CD4+ cells and lead to AIDS (acquired immune deficiency syndrome) when their count is profoundly reduced.

Human immunodeficiency virus (HIV) infection is an important contributor to global disease burden and a leading cause of death. In 2020, the estimated number of persons living with HIV in the world was close to 38 million people; a total of 690,000 people have died from HIV infection and about 1.5 million have been infected this same year [76]. This virus, which targets the immune system, leads to immunodeficiencies and consequently to opportunistic infections and cancers. Due to greater understandings of the virus’s replication cycle, current therapeutic strategies have led to better quality of life for patients, but there is still no effective cure for HIV infection. However, plant peptides could be a novel strategy of HIV infection treatment [77].

**Figure 1:**
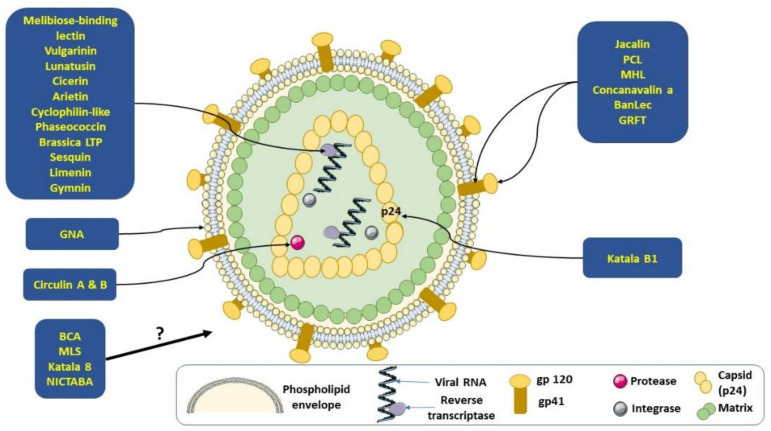
Melibiose-binding lectin, vulgarinin, lunatusin, cicerin, arietin, cyclophilin-like, phaseococcin, brassica LTP, sesquin, limenin, and gymnin inhibit the reverse transcriptase of HIV; circulin A & B inhibit the protease of HIV; katala B1 inhibits p24 capsid protein; jacalin, PCL, MHL, concanavalin a, BanLec, and GRFT inhibit HIV attachment through blocking the viral receptor (gp120/gp41); GNA targets the envelope glycoproteins of HIV-1; BCA, MLS, katala 8, and NICTABA possess anti-HIV activity but the mechanism is not yet elucidated.

Cyclotides belong to a large family of circular, amphipathic, plant-derived peptides characterized by a head-to-tail cyclic backbone and three conserved disulfide bonds. They are numerous and diversified in Violaceae, Rubiaceae, Curcurbitaceae, Fabaceae and Solanaceae [14]. This family presents a number of pharmaceutical potentials and is particularly famous for its quantity of peptides presenting with anti-HIV activity [78]. The position of the disulfide bonds is conserved along the peptide’s sequences, but their topology or activity defines three subfamilies: Möbius, bracelet, and trypsin inhibitors. Their cyclic structure, which provides chemical stability in acidic, high temperature conditions and protection against biological degradation, may allow a *per os* administration. Cyclotides target HIV through interactions with the viral envelope and more specifically by disrupting the viral lipid envelope. This leads to the formation of pores, the leakage of components and the destruction of viral particles before their entry into host cells: cyclotides could prevent fusion between HIV and host cells (Figure 1) [47,51,79].

Kalata B1, which is the most studied and known Möbius cyclotide isolated from leaves of *Oldenlandia affinis*, presents with viricidal activity against HIV. This conclusion is the result of a study in which the peptide had been in contact with two HIV-1 strains (NL4.3/92RW016 using CXCR4 as a co-receptor and a clade A strain using CXCR5 as a co-receptor) at different concentrations. The results showed a 50% reduction in viral infectivity of TZM-bl cells compared with the untreated virus. The half viral infectivity (VC_50_) is 2.04 μM for NL 4.3 and 4.54 μM for Clade A. Another study about the ability of the peptide to disturb HIV-1 particles (strain NL4.3) indicated a decrease in the capsid protein p24 [51]. To evaluate if Kalata B1 targets the membrane to disrupt HIV-1, NL4.3 virus particles were treated with this peptide and results showed a decrease in the viral capsid protein (p24), indicating that Kalata B1 targets the HIV membrane via peptide-lipid interactions (Figure 1) [80].

Kalata B8, a new cyclotide isolated from *Oldenlandia affinis*, provides anti-HIV activity. Its structure appears to be a hybrid between the two major subfamilies of cyclotides. Specifically, loops 2 and 3 of kalata B8 resemble those found in kalata B1 and B2. To determine its antiviral activity, anti-HIV assays were performed in HIV-infected cultured human T-lymphoblast (CEM-SS) cells using the highest 4.5 μM concentration. Results showed that kalata B8 inhibited the cytopathic effects of HIV-1 infected CEM-SS cells with an antiviral cytoprotective concentration (EC_50_) of 2.5 μM, while the cytotoxic concentration for host cells (CC_50_) was higher than 11 μM (Figure 1) [52].

Some plant-derived peptides act by inhibiting HIV-1 reverse transcriptase: among them can be cited the 7 kDa peptides vulgarinin from haricot beans (*Phaseolus vulgaris),* with an IC_50_ of 130 μM [53]; lunatusin isolated from the seeds of the Chinese lima bean (*Phaseolus lunatus* L.), with an IC_50_ of 120 μM [54]; and cicerin and arietin, isolated from chickpea seeds (*Cicer arietinum*), with a molecular weight of approximately 8.2 and 5.6 kDa, respectively. Both peptides possess inhibitory activity toward HIV-1 reverse transcriptase when tested at 200 μM (IC 200 μM) [55]. In addition, cyclophilin-like peptide, isolated from chickpea seeds (*Cicer arietinum*) and characterized by a molecular weight of 18 kDa, is able to inhibit HIV-1 reverse transcriptase with an IC_50_ of about 20 μM [56]. Phaseococcin (5422 Da), isolated from small scarlet runner beans of *Phaseolus coccineus*, possesses anti-HIV activity with an IC_50_ of about 150 μM (Figure 1) [81].

Peptide brassica (LPT) isolated from *Brassica compestris* exhibits an anti-HIV-1 reverse transcriptase activity with an IC_50_ of 4 µM [81,82]. Two peptides without specific names are represented in this list: one of 6 kDa is purified from baby lima beans with an IC_50_ of 4 μM [57] and the other one of 10 kDa originates from the coconut with an IC_50_ value of 52.5 μM (Figure 1) [58].

The defensin-like antimicrobial peptide sesquin (7 kDa), highly homologous to defensin, was isolated from ground beans (*Vigna sesquipedalis)*. An inhibitory activity on HIV-1 reverse transcriptase was determined for this defensin-like peptide, with and IC_50_ between 50 to 200 μM (Figure 1) [83]. Another defensin peptide (5443 Da) without specific designation, isolated from the seeds of the purple pole bean (*Phaseolus vulgaris*) and displaying an IC_50_ value of about 0.5 ± 0.1 μM, proved to be significantly promising compared to other defensins, presenting an average IC_50_ value superior to 40 μM [84]. Some other defensin-like peptides inhibit HIV-1 reverse transcriptase. This is the case for gymnin (6.5 kDa), isolated from the seeds of the Yunnan bean and presenting an IC_50_ value of 200 μM [85], and limenin (6.5 kDa), isolated from the seeds of the shelf bean and presenting an IC_50_ value of 106 µM (Figure 1) [36].

Evidence proved that two macrocyclic peptides named cirulin A and cirulin B, isolated from *Chassalia parviflora* (Rubiaceae), have the capacity to inhibit HIV protease. For both compounds, the antiviral cytoprotective concentrations ranged from about 40 to 260 nM (Figure 1) [59].

Another peptide family, lectins, which are sugar binding proteins that can trigger signals through cell surface molecules involved in lymphocyte activation, has proved its anti-HIV activity [18]. It has been shown that jacalin, a 50 kDa plant lectin purified from jackfruit seeds (*Artocarpus heterophyllus*), has potent antiviral effects and inhibits the HIV-I life cycle at a step before fusion, and also binds CD4 T lymphocytes [21]. *Canavalia ensiformis* lectin, another peptide that belongs to the same family, has the ability to bind gp120 from HIV-1 and HIV-2 isolates, and consequently block the HIV replication cycle [86]. Mannose-specific lectins (MSLs) isolated from the bulbs of fifteen wild Narcissus species were assayed for their HIV-1 infection inhibitory activity in MT-4 cells. It has been shown that this kind of lectin exhibits anti-HIV activity with an EC_50_ of 2.02 μg/mL at 53.7 nM [25]. The sensitivity of *Galanthus nivalis* lectin (GNA) studied against the envelope glycoproteins of HIV-1 showed that GNA could be a potent anti-HIV agent, with IC_50_ values ranging between 1.3 ± 0.2 to >500 nM (Figure 1) [87]. *Polygonatum cyrtonema* lectin (PCL), a novel anti-HIV mannose-binding lectin from the *Galanthus nivalis* agglutinin (GNA)-related lectin family, is characterized by its unique super structure of the PCL mannoside complex. This structure may destabilize the binding of the HIV surface envelop glycoprotein gp120 to specific receptors on cell membranes and block viral fusion [26]. *Myrianthus holstii* lectin (MHL), extracted from the African plant *Myrianthus holstii*, has anti-HIV activity, with an EC_50_ value of 150 nM. This lectin binds a soluble form of the viral envelope protein gp120 [24]. In vitro assays of potent anti-HIV activity of the complete amino acid sequence of a lectin isolated from the green alga *Boodlea coacta* (BCA) was determined by conventional MTT assay using MT-4 cells. Data showed that BCA inhibited HIV-1 infection dose-dependently, with an EC_50_ of 8.2 nM [27]. A dimeric 64-kDa melibiose-binding lectin was isolated from the seeds of *Bauhinia variegate* and manifested potent inhibition of HIV-1 reverse transcriptase activity with a low IC_50_ of 1.02 µM [60]. A unique lectin (NICTABA) produced by the tobacco plant, *Nicotiana tabacum*, has been evaluated for its inhibitory activity against HIV using cell-line-adapted HIV-1 strains. Results revealed that NICTABA was able to inhibit infection by HIV strains with an EC_50_ in the lower micromolar range (EC_50_ 0.023–0.28 mM) [61]. Banana lectin (BanLec) is a jacalin-related lectin isolated from the banana fruit, *Musa acuminata*. This lectin binds to high-mannose carbohydrate structures, the same as those found in viruses containing glycosylated envelope proteins such as HIV-1. It was demonstrated that BanLec might inhibit HIV-1 and laboratory-adapted HIV-1 isolates of different tropisms and subtypes through binding of the glycosylated HIV-1 envelope protein gp120, thus blocking entry of the virus into the cell. This lectin possesses potent anti-HIV activity, with IC_50_ values ranging between 2.06 nM to 0.49 nM (Figure 1) [20].

Studies carried out on peptides extracted from aquatic plants have shown the potential antiviral activity of these compounds. Griffithsin (GRFT), a novel anti-HIV protein, was isolated from an aqueous extract of the red alga *Griffithsia* sp. GRFT displayed antiviral activity against laboratory strains and primary isolates of HIV-1 with EC_50_ values ranging between 0.043 to 0.63 nM. GRFT aborts cell-to-cell fusion and transmission of HIV-1 infection by blocking gp120 binding to receptor-expressing cells and binds to viral surface glycoproteins (gp120, gp41, and gp160) in a glycosylation-dependent manner (Figure 1) [31].

### 3.2. Influenza Strain A H1N1

The influenza A H1N1 virus belongs to the family Orthomyxoviridae, and is an enveloped virus and negative-sense RNA virus. Type A is one of the three major types of influenza viruses, which also include types B and C. Type A is divided into subtypes, which are differentiated mainly on the basis of two surface antigens: hemagglutinin (H) and neuraminidase (N). The H1N1 subtype is further differentiated into strains based on minor variations in RNA sequence [88].

Influenza virus, subtype H1N1, causes moderate to severe respiratory disease and affects all age groups. It was declared as pandemic by the WHO in 2009 [89]. Among influenza A viruses that infect humans, three major subtypes of hemagglutinins (H1, H2, and H3) and two subtypes of neuraminidases (N1 and N2) have been described [90]. The influenza 1 virus has a significant ability to undergo periodic changes in the antigenic characteristics of their envelope glycoproteins, the hemagglutinin and the neuraminidase, and consequently, it presents a novel circulating strain resist to eventual vaccination as well as treatments [91]. The only two classes of antiviral drugs against influenza are the adamantane and neuraminidase inhibitors, for which there are already described cases of resistance [91]. Natural remedies and plant peptides have been described to have antiviral activity against the influenza H1N1 virus.

Cyclotides from different plant species have been significantly investigated for their ability to inhibit human viruses, including influenza H1N1 [13]. A cyclotide isolated from the whole plant of *Viola yedoensis*, cycloviolacin VY1, was previously reported as having antiviral activity against the H1N1 virus. Experimentation showed that the IC_50_ value of cycloviolacin VY1 against influenza A H1N1 virus was 2.27 ± 0.20 mg/mL. This was the first cyclotide described as an anti-influenza A agent against the H1N1 virus [62].

In addition, it was described that a blood glycoprotein fetuin-binding peptide LTP, with a molecular mass of about 9 kDa, isolated from the bulbs of the Chinese daffodil, *Narcissus tazetta var. chinensis L*., has the ability to inhibit influenza A H1N1 virus replication. Studies suggest that this antiviral activity can be related to the capacity of fetuin to bind the virus and consequently to block the neuraminidase in the viral envelope [63]. The half maximal effective concentration of this peptide LTP (EC_50_) that inhibits 50% of the replication of influenza A H1N1 virus is about 4.47 mg/mL, determined by MTT assay, a colorimetric assay for assessing cell metabolic activity and viability, used in this study to evaluate the survival rate of Madin–Darby Canine Kidney (MDCK) cells [64]. Another Narcissus tazetta lectin (NTL) exhibits evenly strong antiviral properties against influenza A (H1N1) with IC_50_ values ranging between 0.20 µg/mL to 1.33 µg/mL in a dose-dependent manner. It is expected that the antiviral mechanism of NTL against influenza A virus is achieved mainly through acting on the early stage of the viral cycle of influenza A (H1N1) virus [65].

*Yucca filamentosa* lectin (YFL-I) is part of the *Galanthus nivalis* agglutinin (GNA)-related lectin family, and also exhibits anti-influenza activity. Accordingly, per the results of the study of Xi et al., *Yucca filamentosa* lectin, as GNA-related lectins, could prevent influenza virus infection. YFL-I has a higher affinity than influenza hemagglutinin to bind host cell sialic acid, and this may block the early stages of viral entry and infection [23].

A lectin isolated from the green alga *Boodlea coacta* (BCA) showed its potent activity against different influenza virus strains by directly binding to viral envelope hemagglutinin. Experimentations were made on MDCK cells by the neutral red dye uptake assay, which provides a quantitative estimation of the number of viable cells in a culture. Ten influenza A virus strains, including laboratory-adapted strains as well as one influenza virus B strain, were used in the study to determine the antiviral activity of BCA. Data showed that BCA has a stronger capacity to inhibit H3N2 subtypes at EC_50_ values of 18.8–74.2 nM, while the antiviral activity of BCA was much weaker against H1N1 subtypes demonstrated by the EC_50_ values of 79.3–1590.2 nM. The clinical isolates of the pandemic strain, swine-origin influenza virus, A/Oita/OU1 P3-3/09 (H1N1), were also susceptible to BCA. This study also determined that BCA directly binds to the oligosaccharide on the influenza envelope glycoprotein hemagglutinin. For that, enzyme-linked immunosorbent assays (ELISA) were performed using an influenza vaccine preparation, which contains hemagglutinin of A/California/7/09 (H1N1), A/Victoria/210/09 (H3N2), and B/Brisbane/60/08 influenza strains. Results indicate that H3N2 subtypes are more sensitive to 1–2-linked mannose-binding lectins than H1N1 subtypes [27].

### 3.3. Junin Hemorrhagic Fever Virus (JHFV)

JHFV is an enveloped virus containing two single-stranded RNA molecules. Virions are pleomorphic; particles vary in diameter from between 40 to 200 nm. Around 5 million humans run the risk of being infected by the Junin virus, which causes a hemorrhagic fever. Described for the first time in the 1950s, recent progress on JHFV-induced pathology and on the virus itself is leading to the development of potential vaccines and drugs [92].

The meliacine peptide, described above for its anti HSV-activity, is also efficient against the Junin hemorrhagic fever virus. It targets the early step of virus infection by preventing the uncoating step, disturbs the release of viral particles and interferes with the low-pH-induced fusion of infected cells [49].

### 3.4. Respiratory Syncytial Virus (RSV)

Respiratory syncytial virus (RSV) is a member of the Paramyxoviridae family and contains a single-stranded negative-sense RNA genome. The RSV genome encodes for 11 structural and nonstructural proteins, including the nucleoprotein, phosphoprotein and RNA-dependent RNA polymerase encapsulate the viral RNA. This structure forms the minimal replication machinery. RSV possesses three integral membrane proteins: the receptor attachment glycoprotein, the fusion protein, and a short hydrophobic protein [93].

In 2017, according to the World Health Organization (WHO), RSV remains the most common cause of lower respiratory tract infection in neonates and young infants, especially in low- and middle-income countries [94]. Nowadays, prevention in premature or severely immunosuppressed infants relies on palivizumab, a monoclonal antibody with a rather high risk of side effects (cardiac and pulmonary) [94,95]. Ribavirin’s application in RSV infection has not showed a significant advantage in clinical usage (decrease in mortality, in hospitalization duration, intensive care unit admission and in mechanical ventilation needs) and is no longer recommended [95,96]. The RSV vaccine roadmap priority targets immunization of young infants [96]. Effectively, it is supposed that young children (<10 years old) could play a major role in RSV propagation [97]. In developed countries, RSV also infects young infants (<3 months years old), with a seasonal peak during November and December [98]. However, RSV infection can equally occur in elderly individuals (especially in long term care facilities), suggesting that RSV can be an important cause of morbidity [94]. As of 2021, no vaccine has yet been developed [99].

Development of new RSV disease treatments should focus on improvement in symptoms, clinical expression reduction, and transmission reduction. Several new potential drugs have been evaluated in clinical trials (phase 1 and 2), but it seems that none of them are from plant origin. For example, RSV521 or sisunatovir showed 63% virus load reduction at the dose of 350 mg per os and seems promising. It was developed by drug design optimization [100,101]. Ziresovir is currently undergoing a phase 2 double-blind clinical trial in the adult population [101]. However, the need for new preventive and therapeutic drugs against RSV infections remains strong. Vaccination remains the main objective against RSV pandemics. There are 3 protein-based vaccine candidates (fusion inhibitors) in phase 3 clinical trials so far [102]. Two are from GlaxoSmithKline (one for maternal prevention and one for elderly people), while the third one is from Pfizer and targets maternal prevention [102].

Other biological resources and strategies, such as plant peptide antivirals, may help to control the spread of this disease [99]. However, regarding the natural products obtained from plants, few molecules have been identified so far. Fetuin-binding peptide LTP, which inhibits replication of H1N1 virus, also showed antiviral activity against RSV [63,64]. This study was performed in RSV-infected HEp-2 cell monolayers; antiviral assay demonstrated that LPT peptide could reduce 37.55% of plaque formation by RSV at a final concentration of 50 μg/mL [64]. Furthermore, it could block the entrance of the RSV in host cells [64]. Narcissus tazetta lectin (NTL) also showed the capacity to inhibit plaque formation by the human RSV with an IC_50_ of 2.30 µg/mL during the whole viral infection cycle [65].

### 3.5. Flavivirus (FV)

The genus flaviviruses, from the Flaviviridae family (*flavus* is the latin word for yellow), are positive single-strand RNA viruses that infect mammals. The genome (about 10 kb) codes 3 structural proteins (C, prM, E) and 7 nonstructural proteins (NS1 to NS7). The viral particle size is about 50 nm. Yellow fever virus (YFV), which has given the name to the genus, was first discovered in the 1920s. It was followed by the West Nile virus (WNV), Japan encephalitis virus (JEV), tick-borne encephalitis virus (TBEV), DENV and ZIKV, which are all part of the flaviviruses. They represent a major health concern all over the world, causing emergent and re-emergent infectious diseases. Mosquito species as *Aedes* sp. and *Culex* sp. are the main vectors, except for TBEV [103].

Flu-like syndromes are the predominant symptoms. However, complications (articular, encephalitis, neuropathology, and organ failure) may occur and lead to high death rates. To date, no specific treatment is available in most cases. Treatment relies mostly on nursing, hydration, and complication prevention. Vaccines are available for some viruses and remain the main prevention strategy, coupled with vector control [104]. Several drug-developing strategies against FV have been discussed. For instance, the review of Boldescu et al. proposes that anti-flavivirus drug development should focus on targeting NS3 and NS5. They also mention an interest in broad-spectrum activity regarding the structural proximity of Flaviviridae [105].

Nowadays, little to no information is available on the anti-flavivirus activity of plant-derived peptides, with few exceptions.

#### 3.5.1. Yellow Fever Virus (YFV)

Yellow fever virus (YFV) is a member of the family Flaviviridae, genus *Flavivirus*. The YFV genome is a single-stranded positive-sense RNA genome coding for a polyprotein, which is processed into three structural proteins and seven non-structural proteins. The major component of the virion surface is the envelope (E) protein and membrane (M) protein. It is the primary immunogen and plays a central role in receptor binding and membrane fusion [106]. There are 70 serotypes of YFV, which are transmitted by mosquitos (*Aedes* and *Haemogogus* species) and ticks.

Yellow fever is currently endemic in tropical regions of Africa and South America, where sporadic cases, limited outbreaks, or large epidemics are periodically detected on both continents [107].

Efficient vaccines (Stamaril, YF-VAX, 17D-YFV) with an attenuated virus are available, but epidemics continue to spread in new areas due to population movement.

The risk-versus-benefit of vaccinating at-risk immunocompromised persons (e.g., HIV-infected, rheumatoid arthritis patients under treatment with disease-modifying anti-rheumatic drugs, DMARD) against YFV poses numerous challenges, leading to the question of whether withholding YF vaccination for immunocompromised travelers is reasonable [108].

According to the PanAmerican Health Organization, a WHO subdivision, YFV infects 200,000 people each year, leading to 30,000 deaths, resulting in a mortality of 15% [107].

Monkeys are the main reservoir of the virus and mosquitos are the vectors. Humans (e.g., forest workers) can be infected through mosquito bites, which leads to sporadic cases.

Larger epidemics occur when a virus is introduced into an urban area with a high human and mosquito population density. Lack of vaccination or prior exposure to YFV could lead to the worst-case scenario for YFV epidemics. During the infection, prevention and treatment of dehydration, as well as prevention of kidney and liver failure, increase the survival rate [109].

According to Fiegueiredo et al., a steroidal saponin from *Solanum sisylbriifolium* (ethanolic extract) may have an inhibitory activity against DENV and YFV [110].

Two report studies dating from 2003 and 2009 show that essential oils from aromatic plants of San Luis, Argentina, along with citral, limonene and essential oils from *Lippia citriodora* and *L. albahave* present antiviral activity against YFV. No plant-derived peptides have been described yet as potential anti-YFV agents [111]. The question whether plant-derived peptides can play a potential role as anti-YFV is still under investigation.

#### 3.5.2. Dengue Virus (DENV)

DENV is an enveloped virus with a single positive-stranded RNA of the family Flaviviridae. The DENV genome codes for three structural proteins (capsid protein C, membrane protein M, envelope protein E) and seven nonstructural proteins (NS1, NS2a, NS2b, NS3, NS4a, NS4b, NS5) [112].

Each year, between 100 and 400 million humans are infected by this virus, transmitted by female mosquitoes of Aedes species (*Ae.* *aegypti* or *Ae. albopictus*) in countries with a subtropical and tropical climate. Four different viral serotypes exist and forms of infections can vary from subclinical to severe dengue with life-threatening situation. The disease is currently incurable and the incidence of novel infections has increased in recent years [113].

The sequence of the cyclotide kalata B1, known for its anti-HIV activity as described above, has been modified to present an anti-DENV activity. Called “kalata B1-inspired peptide”, it inhibits dengue NS2B-NS3 protease, which leads to aborted viral replication [28].

Two peptides, called peptides 2 and 4, derived from the Asian medicinal plant *Acacia catechu* (Fabaceae—originally found in Asia) present an anti-DENV activity by targeting the early steps of the viral entry phase. The study of Panya et al. demonstrates that these two peptides at a concentration of 50 μM stop more than 90% of DENV foci formation, defined as localized clusters of infected cells. Their IC_50_ value is 0.18 μg/mL. According to the authors, 1.25 μg/mL of peptides results in a 100-fold virus production reduction without toxicity to cells [66].

Another study based on a synthetic bioactive peptide extracted from the same plant confirmed its efficiency against the four serotypes of DENV. Further investigations with this peptide, called Pep-RTYM, showed that it targets the early step of virus infection, similar to the kalata B1-inspired peptide. Incubation of Pep-RTYM with the virus before infection of the host cells results in more effective inhibition than incubation with the peptide during or after infection of the host cell with DENV. Binding assays led to the conclusion that PEP-RYTM binds viral particles. Moreover, it also inhibits the replication and production of novel viral particles in a dose-dependent manner [66].

Cystine knot α-amylase inhibitors, also called alstotides, are cysteine- and proline- rich peptides derived from Amaranthaceae and Apocynaceae species. *Alstonia scholaris* contains the As1 peptide, which has been added to cells at the same time as the DENV serotype 2. The expression of nonstructural viral protein 3 (NS3) after 3 days of incubation with the peptide is reduced and the EC_50_ value is close to 90 μM [67].

#### 3.5.3. Japan Encephalitis Virus (JEV)

Japanese encephalitis virus (JEV) is a member of the flavivirus family. Its positive-sense single-strand RNA genome codes for three structural proteins: Capsid (C), membrane (prM/M) and envelope protein (E), and seven non-structural (NS) proteins: NS1, NS2A, NS2B, NS3, NS4, NS4B, and NS5. The external envelope of the virus contains E and M proteins that play a role in host cell invasion. The virus contains also a nucleocapsid which is composed of multiple copies of the capsid protein, enclosing the nucleic acid [114].

JEV is the main cause for arbovirus encephalitis worldwide. It is an endemic virus widely distributed in Asia, including Japan, China, Taiwan, Korea, the Philippines, far-Eastern Russia, Southeast Asia, India, Papua New Guinea, and the Torres Strait of Australia. As in the majority of viral infections, there is no specific antiviral treatment for JEV infections; there is only supportive care and management of the clinical complications.

Only one vaccine has been authorized for JEV infection, based on inactivated viral particles. However, it remains expensive, and antibody protection is not conserved on a long term, requiring multiple administrations. Recombinant vaccines are currently being developed and are at the clinical trial stage [103].

A study conducted by Navyashree et al. aimed to evaluate the docking potential of phytoconstituents (i.e., perolyrine, 5,11 Dihydroindolol [3, 2-ß] carbazole, 1-methoxycarbonyl-β-carboline, 1-(β-carboline-1-yl)-3,4,5-trihydroxy-1-pentanone and (*S*)-1-(1′-hydroxyethyl)-β-carboline) of the *Arisaema* genus. The authors concluded that pyroline and 5,11-Dihydroindolo [3, 2-ß] carbazole interact with viral proteins such as NS3 and NS5 helicase, presenting good pharmacokinetics and safety profiles. However, further analyses are needed to confirm the antiviral activity [115].

In vitro experiments showed that treatment of JEV with griffithsin (GRFT), a lectin isolated from the red marine alga Griffithsia sp., in BHK- 21 cells inhibited JEV with an IC_50_ of 20 nM. In vivo experiment showed that GRFT (5 mg/kg) administered intraperitoneally could prevent mortality in mice challenged intraperitoneally with a lethal dose of JEV. Further research is required to elucidate the mechanism by which GRFT exerts its anti-JEV activity. As in other viruses such as severe acute respiratory syndrome coronavirus (SARS-CoV) [32], and HIV-1 [87], it was found that GRFT binds to glycosylated viral proteins to inhibit infection [33].

#### 3.5.4. Tick-Borne Encephalitis Virus (TBEV)

Among flaviviruses, tick-borne encephalitis virus (TBEV) contains a spherical, enveloped capsid. It has a long positive-strand RNA genome that encodes for ten main viral proteins. The three structural proteins (SP) consist of capsid protein (C), viral envelope (E) and membrane (M) proteins. The seven non-structural proteins (NS) (NS1, NS2A, NS2B, NS3,NS4A, NS4B and NS5) have a role in the replication machinery of the viral genome [116].

Tick-borne viruses regroup the TBE complex (8 viruses, including TBEV), which infect mammals, and the Tyuleniy group (3 viruses), which infects seabirds. Three subtypes of TBEV are known depending on their geographical origins (European, Siberian, and Far Eastern). Ticks from the *Ixodes* genus are the principal vector and reservoir of the virus. Person-to-person transmission has not been reported, but vertical transmission from infected mother to fetus has been described. Infection can also occur by drinking raw milk from infected goats, sheep, or cows. Between 10,000 to 15,000 cases of TBEV infection are reported in Asia and in Europe each year. The best prevention against TBEV remains vaccination [116].

#### 3.5.5. Zika Virus (ZIKV)

ZIKA Virus (ZIKV) is one of the enveloped viruses. It is a positive-sense, single-stranded RNA virus, encoding three structural proteins: capsid (C); membrane (M); and envelope (E) and seven 7 nonstructural proteins (NS1, NS2A, NS2B, NS3, NS4A, NS4B, and NS5) that assist in replication of the genome and packaging of the virion [117].

In the period between 2007 and 2013, ZIKV revealed its epidemic potential with more than 20,000 infected people. There are no specific treatments nor a vaccine. Treatment is mostly symptomatic with acetaminophen and antihistaminic molecules [117,118].

It has been reported that several promising plant-based products including peptides have shown activity against ZIKV, especially when NS2B and NS3 viral proteins are targeted, and could be used for drug design [104].

#### 3.5.6. Chikungunya Virus (CHIKV)

Chikungunya virus (CHIKV) is an emerging mosquito-transmitted pathogen belonging to the *Alphavirus* genus of the Togaviridae family. Their structure consists of enveloped icosahedral capsids, which contain a single-stranded positive RNA, encoding five structural proteins (capsid [C], E3, E2, 6K/TF, and E1) responsible for the production of infectious virions; and four nonstructural proteins (nsP1, nsP2, nsP3 and nsP4) that are implicated in viral replication and transcription processes [119].

CHIKV was reported firstly in Tanzania in 1952 and emerged as an epidemic in 2005 in the French island of Réunion, then spread in different countries including Asia, Europe and Africa. This infectious disease causes high fever, headache, rashes, myalgia, arthralgia, and crippling arthritis that may persist for a long time. Other severe symptoms, including encephalitis, hemorrhagic disease, and mortality, have also been reported during recent epidemics [119]. Currently, there are no licensed vaccines or antivirals available against CHIKV infection [120].

Chitinase (chi)-like lectin, isolated from *Tamarindus indica* (TCLL), showed antiviral activity against CHIKV. Experimentation was performed using BHK-21 (cells derived from baby hamster kidney) and Vero cells. It was demonstrated that CHIKV viral RNA levels upon treatment with 100 μM lectin were reduced to nearly 45% [69].

#### 3.5.7. Hepatitis C Virus (HCV)

Hepatitis C virus (HCV) is composed of an icosahedral enveloped capsid with a positive single-stranded RNA. It belongs to the Flaviviridae family, that contains viral envelope glycoproteins, E1 and E2, which are embedded in the lipid envelope and implicated in the invasion into the host cell [121]. HCV enters into hepatocytes and causes cell necrosis leading to a systematic viral infection that can be acute or chronic [122].

HCV infection is a public health problem with a global prevalence up to 2.8% of chronic infections [122]. According to the WHO, infection with HCV does not always require treatment, particularly in immunocompetent persons. However, HCV infection should be treated in all patients with chronic HCV infection over the age of 12. Only pan-genotypic direct-acting antiviral (DAAs) treatment remains effective, but it is expensive in many high- and upper-middle-income countries [123,124]. It was reported that GRFT lectin has the most potent anti-HCV activity with an EC_50_ value of 0.4 nM. GRFT showed low toxicity exhibiting a 50% cytotoxic concentration (CC_50_) equal to 34 mM [34]. 

### 3.6. Infectious Bronchitis Virus (IBV)

IBV is a gammacoronavirus (RNA simple strand, positive sense, enveloped virus) that infects birds, mainly chickens. The IBV genome is very diverse in nature and numerous serotypes exist; this is probably due to the recombination between two or more strains during coinfection in chickens and to the low fidelity rate of the viral RNA-dependent RNA polymerase (RdRp). The disease, which is highly contagious, principally damages the upper respiratory system followed by the loss of ciliature activity due to viral fixation on sialylaminoglycan on the epithelium, leading to respiratory distress. The virus-damaged epithelium can thus enhance coinfection with bacteria. The kidneys and reproductive system can also be infected [125,126]. 

The presence of different viral serotypes, which do not cross-react with IBV infection-induced antibodies, leads to difficulties in controlling the spread of the viruses.

According to Jackwood (2012) and Bande (2017), at least 10 types of IBV are frequently isolated in the USA, with several variants for each type, which means the viruses continue changing and evolving. The same review mentions at least 8 different types for Europe, China, and 4 for Korea, Israel, and Japan, each, illustrating the great diversity of the virus [125,127]. 

According to Cavanagh, IBV is the main infectious disease with major economic loss in several countries due to the hundreds of IBV serotypes, differing mostly in one of the two viral spike surface glycoproteins (S1) structure by about 20–25% [128].

Vaccination remains the main strategy against IBV, with several challenges due to the variants and the diversity of serotypes [129].

Some peptides from other origins have been reported to have an antiviral action [130]. One such example is knottins—antimicrobial peptides containing 3 disulfide bonds, with many members in plants, but also present in other eukaryotes. Alstotides are knottins from the blackboard tree *Alstonia scholaris*. Parthasarathy reported the anti-IBV activity of one alstotide, As1, which functions by blocking the spike protein and thus inhibiting the fusion of the virus with the host cell during the viral replication cycle. The same knottin may also interact with the IBV membrane (M) protein and consequently present a multitarget activity [68].

The examples of antiviral plant peptides remain few, but IBV is an epidemiological and economic threat to avian industries. Thus, IBV’s molecular diversity and continuous tendency of emerging new variants, combined with its proximity to the human population, should encourage the research of plant-based strategies as plants remain an interesting source of bioactive molecules, including peptides.

### 3.7. Foot and Mouth Disease Virus (FMDV) and Coxsackie Virus

Foot and mouth disease virus (FMDV) has a capsid enclosing a positive single-stranded RNA. The FMDV genome encodes for four structural polypeptides (VP1 to VP4) and a nonstructural protein (NSP) that help the virus to evade the host immune mechanisms and cause an aggressive infection [131]. FMDV is a very contagious pathogen that affects a wide range of cloven-hoofed animals [132]. FMDV contains seven serotypes (types O, A, C, and Asia and the South African territories types 1, 2 and 3) [133]. Meliacine, a peptide which targets the replication cycle of HSV-1, was described as having an inhibitory effect on FMDV attachment to the cell surface. Meliacine inhibits FMDV virulence in BHK-21 monolayers in a dose-dependent manner, with an the EC_50_ of 0.5 mg/mL [50]. In addition, a lectin was purified from the rhizomes of Aspidistra elation Blume, named A. elatior lectin (AEL); it appears to have antiviral activity against Coxsackie Virus B4 with an IC_50_ of about 4 μg/mL [70].

### 3.8. Ebola Virus

Ebola virus disease (EVD) constitutes the family Filoviridae within the order Mononegavirales. It contains an RNA genome and seven viral proteins implicated in replication, transcription and viral assembly: NP, VP35, VP40, GP (glycoprotein), VP30, VP24, and an RNA-dependent RNA polymerase (L) [134].

EVD is a fatal syndrome in humans, with average case fatality rates of around 50%. It causes gastrointestinal symptoms such as severe watery diarrhea, nausea, vomiting, abdominal pain and fever [135]. Currently, there is no licensed vaccine or treatment for EVD [136]. It has been previously reported that engineered banana lectin (BanLec) has an antiviral activity against viruses including Ebola virus. BanLec inhibited both entry and replication of Ebola virus in HEK293T/17 (human embryonic kidney) cell cultures, with IC_50_ values of about 1 ± 6 μM [71].

### 3.9. Coronavirus

The coronavirus belongs to the family Coronaviridae, order of Nidovirales and the suborder of Coronavirineae. It contains a positive-sense single-stranded RNA polymer surrounded by a protective capsid, which contains nucleocapsid (N). The coronavirus particle is surrounded by an outer membrane envelope made of lipids with viral proteins inserted, including the spike (S), membrane (M), and envelope (E) proteins [137].

The severe acute respiratory syndrome coronavirus (SARS-CoV) is a strain of coronavirus that causes severe respiratory syndromes [138]. This infection spread rapidly and results in the appearance of a pandemic as it was reported in the early months of 2020. The WHO announced a novel SARS-CoV- 2 strain, named coronavirus disease 2019 (COVID-19), which has developed into a global pandemic since March 2020 [139]. Moreover, 2012 SARS-CoV caused a pandemic named Middle East respiratory syndrome (MERS)-CoV, reported particularly in the Middle East region [140]. No specific effective treatment has been clinically developed. Several medical companies are working on antiviral drugs, but to date, only some vaccines are used as a preventive measure against the disease, while others are still being validated or tested [141]. Some studies evaluated the antiviral effect of griffithsin (GRFT), a lectin isolated from the red marine alga Griffithsia sp., against SARS-CoV and MERS-CoV infections [19,32,35]. The antiviral study of GRFT against SARS-CoV (Urbani strain, MA15) showed that GRFT lectin potently inhibits the cytopathic effect of SARS-CoV in Vero 76 cells; GRFT reduced the percentage of cells killed by SARS-CoV (Urbani, MA15) in a concentration-dependent manner (EC_50_ 48 nM) [32].

The antiviral effect of GRFT against MERS-CoV infection demonstrated that GRFT inhibits MERS-CoV spike protein function (mediates binding to the host cell surface) during the entry into host cells at 2 mg/mL. Consequently, GRFT could be a potent inhibitor of MERS-CoV infection [35].

Future research efforts focused on the GRFT lectin, and other plant-derived antimicrobial peptides, might provide effective therapeutic options for the treatment of COVID-19 and other viral infections causing debilitating diseases and global pandemics.

## 4. Conclusions

Interest in peptides has been increasing for several years now, in particular for their therapeutic potential in many fields such as viral infections and microbial infections. They have numerous advantages: a broad range of activities, low side effects in the host due to high selectivity, specificity, biocompatibility, ability to penetrate cells, and low toxicity, antigenicity and immunogenicity. However, very scarce information is available on plant peptides with antiviral activity in the existing scientific literature and the current state of knowledge is quite limited on this topic. This is contrasting with other plant-derived molecules (i.e., terpenoids, saponins, or glycans), which have been extensively explored for their anti-infectious properties. Finally, even if plant peptides have not revealed their full potential yet, they remain interesting to explore in the context of emerging and re-emerging infectious diseases. The traditional use of plant-derived drugs remains the principal source of bioactive molecules worldwide. This review underlines the need for further investigations on the mechanisms of action of plant-derived peptides. There is also a need for new formulation strategies to enable the development of new drug candidates and peptide-based antiviral therapies.

## Figures and Tables

**Table 1:** Antiviral activities of plant peptides.

Peptides	Plant Source	Virus Target	EC_50_	IC_50_	References
Cyclotides	Rubiaceae and Violaceae plant families	HIV, infuenza H1N1 and DENV—disruption of viral envelope	-	-	[13] [15]
Kalata B1-inspired peptide	Synthetic derivative of kalata B1 peptide	Anti-DENV NS2B–NS3 protease	NI	Isomer 1B: 4.3 μM Isomer 1C: 9.3 μM	[13] [28]
Griffithsin (GRFT)	*Griffithsia sp (Montagne)*	Anti-HIV, -MERS- CoV, -SARS-CoV, -hepatitis C virus (HCV), -HSV-2, -human papillomavirus (HPV), and anti-Japanese encephalitis virus (JEV)	SARS-CoV: 48 nM HSV2 EC_90_: 12 ng/mL MERS- CoV: 2 mg/mL JEV EC50: 100 µg/mL HCV: 0.4 nM HPV16: 1.39 μM HPV18: 0.428 μM HPV45: 0.928 μM	HIV:0.043–0.63 nM JEV: 20nM	[19] [29] [30] [31] [32] [33] [34] [35]
Peptide with sequence homology to defensins	*Phaseolus vulgaris cv.*	Reduction of the activity of HIV-1 reverse transcriptase	NI	0.5 μM	[13] [20]
Banana lectin (BanLec)	*Musa acuminate cultivars cv. Grand Nain*	Anti-HIV activity	NI	0.49–2.06 nM	[20]
*Artocarpus heterophyllus* (jacalin) lectin	*Artocarpus heterophyllus* Lam.	Anti-HIV activity	NI	NI	[21]
Galanthus nivalis (snowdrop) agglutinin-related lectins *Yucca filamentosa* lectin (YFL-I)	*Galanthus nivalis* L.	Anti-HIV activity; antiviral activity against influenza virus H1N1	NI	HIV: >500 nM	[23] [36]
Myrianthus holstii lectin (MHL)	Myrianthus holstii Engl.	Anti-HIV activity	150 nM	NI	[24]
Narcissus pseudonarcissus lectin (NPA)	*Narcissus pseudonarcissus* *L.*	Anti-HIV activity	2.02 μg/mL	53.7 nM	[25]
*Polygonatum cyrtonema* lectin (PCL)	*Polygonatum cyrtonema (Hua)*	Anti-HIV activity.	NI	NI	[26]
Boodlea coacta lectin (BCA)	Boodlea coacta (Dickie)	Anti-HIV activity; antiviral activity against influenza virus H1N1	HIV: 8.2 nM H3N2: 18.8–74.2 nM H1N1: 79.3–1590.2 nM	NI	[27]
Phaseococcin	*Phaseolus coccineus (Minor)*	Inhibition of the reverse transcriptase activity of the HIV virus	NI	150 μM	[10] [20] [22] [37]
Sesquin (defensin-like)	*Vigna sesquipedalis* *cv. ‘Ground Bean’*	Inhibition the reverse transcriptase activity of the HIV virus	NI	50–200 μM	[38] [39]
Limenin (defensin-like)	*Phaseolus limensis cv.*	Reduction of the activity of HIV-1 reverse transcriptase	NI	106 µM	[40]
Gymnin	*Gymnocladus chinensis Baill.*	Inhibition of the reverse transcriptase activity of the HIV virus	NI	200 μM	[41]
2 kD peptide	*Sorghum bicolor (L.) Moench*	Inhibition of the replication of HSV-1	6.25 μM	6.25 to 50 μM	[13] [40] [41] [42]
Meliacine	*Melia azedarach* L.	Antiviral action on HSV-1, Junin virus (JV), and foot and mouth disease virus (FMDV)	HSV: 0.82 μg/mL HSV TK^−^: 0.41 μg/mL JV: 0.13 μg/mL FMDV: 0.5 μg/mL	NI	[43] [44] [45] [46] [47] [48] [49] [50]
*Canavalia ensiformis* Lectin (concanavalin a)	*Canavalia ensiformis* *L.*	Anti-HIV activity	NI	NI	[48]
Pep-RTYM	*Synthetic derived plant peptide*	Anti-DENV activity	1.25 µg/mL		[48]
Kalata B1	*Oldenlandia affnis (Roem. & Schult.)*	Anti-HIV activity by destroying the viral particles prior to cell entry and inhibiting fusion of the virus to the host membrane	2.04 μM	NI	[51]
Kalata B8	*Oldenlandia affinis (Roem. & Schult.)*	Anti-HIV activity, proposed to be membrane dependent	2.5 μM	NI	[52]
Vulgarinin	*Phaseolus vulgaris*	Inhibition of HIV-1 reverse transcriptase	NI	130 μM	[53]
Lunatusin	*Phaseolus lunatus* L.	Inhibition of HIV-1 reverse transcriptase	NI	120 μM	[54]
Cicerin	*Cicer arietinum* L.	Anti-HIV-1 reverse transcriptase activity	NI	200 μM	[55]
Arietin	*Cicer arietinum* L.	Anti-HIV-1 reverse transcriptase activity	NI	200 μM	[55]
Cyclophilin-like	*Cicer arietinum* L.	Inhibition HIV-1 reverse transcriptase	NI	20 μM	[56]
Brassica LTP	*Brassica campestris ssp. chinensis*	Inhibition of the activity of HIV-1 reverse transcriptase	NI	4 µM	[57] [58]
Circulin A & B	*Chassalia parvifolia (K. Schum.)*	Anti-HIV activity	NI	40–260 nM	[59]
Melibiose-binding lectin *Bauhinia variegata* lectin	*Bauhinia variegate* L.	Inhibition of the reverse transcriptase activity of the HIV virus	NI	1.02 µM	[60]
Lectin (NICTABA)	*Nicotiana tabacum var. Samsun NN*	Anti-HIV activity	0.023–0.28 mM	NI	[61]
Cycloviolacin VY1 and VY5	*Viola yedoensis (Makino)*	Anti-HIV activity Anti-influenza A H1N1 virus	NI	2.27 mg/mL	[62]
Fetuin-binding peptide (lipid transfer proteins-LTP)	*Narcissus tazetta var.* *chinensis*	Inhibition of RSV and the cytopathic effect induced by influenza A (H1N1) virus	H1N1: 4.47 μg/mL RSV: 50 μg/mL (final C)	NI	[63] [64]
Narcissus tazetta lectin [NTL]	*Narcissus tazetta var.* *chinensis*	Inhibition of RSV and the cytopathic effect induced by influenza A (H1N1) virus	RSV: 2.30 µg/mL H1N1: 0.20 µg/mL	NI	[65]
Peptide 2 and 4	*Acacia catechu (L.f.) Willd*	Anti-DENV	6.25 μM	15.25 μM	[66]
Knottins Alstotides (As1)	*Alstonia scholaris Linn. R. Br.*	Inhibition of the early phase of infectious bronchitis (IBV) (binds to IBV spike) virus and DENV infection	90 μM	NI	[67] [68]
*Tamarindus indica lectin* (TCLL)	*Tamarindus indica* L.	Anti-alphaviruses (chikungunya viral infection)	>100 µM	N	[69]
Lectins	*Aspidistra elatior (Blume)*	Antiviral action on Coxsackie virus B4 and RSV	4 μg/mL	100 μg/mL	[70]
Engineered banana lectin (BanLec)	*Synthetic derived plant peptide*	Antiviral activity against Ebola virus disease (EVD)	NI	1–6 µM	[71]

## Data Availability

Data sharing not applicable.
